# Integrating evidence-based practices for increasing cancer screenings in safety net health systems: a multiple case study using the Consolidated Framework for Implementation Research

**DOI:** 10.1186/s13012-016-0477-4

**Published:** 2016-08-02

**Authors:** Shuting Liang, Michelle C. Kegler, Megan Cotter, Phillips Emily, Derrick Beasley, April Hermstad, Rentonia Morton, Jeremy Martinez, Kara Riehman

**Affiliations:** 1 Emory Prevention Research Center, Department of Behavioral Sciences and Health Education, Rollins School of Public Health, Emory University, 1518 Clifton Road NE, Atlanta, GA 30322 USA; 2 Statistics and Evaluation Center, Department of Intramural Research, American Cancer Society, Inc., 250 Williams St., Atlanta, GA 30303 USA

## Abstract

**Background:**

Implementing evidence-based practices (EBPs) to increase cancer screenings in safety net primary care systems has great potential for reducing cancer disparities. Yet there is a gap in understanding the factors and mechanisms that influence EBP implementation within these high-priority systems. Guided by the Consolidated Framework for Implementation Research (CFIR), our study aims to fill this gap with a multiple case study of health care safety net systems that were funded by an American Cancer Society (ACS) grants program to increase breast and colorectal cancer screening rates. The initiative funded 68 safety net systems to increase cancer screening through implementation of evidence-based provider and client-oriented strategies.

**Methods:**

Data are from a mixed-methods evaluation with nine purposively selected safety net systems. Fifty-two interviews were conducted with project leaders, implementers, and ACS staff. Funded safety net systems were categorized into high-, medium-, and low-performing cases based on the level of EBP implementation. Within- and cross-case analyses were performed to identify CFIR constructs that influenced level of EBP implementation.

**Results:**

Of 39 CFIR constructs examined, six distinguished levels of implementation. Two constructs were from the intervention characteristics domain: adaptability and trialability. Three were from the inner setting domain: leadership engagement, tension for change, and access to information and knowledge. Engaging formally appointed internal implementation leaders, from the process domain, also distinguished level of implementation. No constructs from the outer setting or individual characteristics domain differentiated systems by level of implementation.

**Conclusions:**

Our study identified a number of influential CFIR constructs and illustrated how they impacted EBP implementation across a variety of safety net systems. Findings may inform future dissemination efforts of EBPs for increasing cancer screening in similar settings. Moreover, our analytic approach is similar to previous case studies using CFIR and hence could facilitate comparisons across studies.

**Electronic supplementary material:**

The online version of this article (doi:10.1186/s13012-016-0477-4) contains supplementary material, which is available to authorized users.

## Background

Although cancer mortality rates in the USA have been declining, significant disparities persist, especially by race/ethnicity and socio-economic status [1–3]. Unfortunately, disparities also exist in early detection through cancer screening [3–5]. The persistence of these disparities warrants targeted efforts to employ evidence-based practices (EBPs) within settings that reach populations experiencing a disproportionate share of the cancer burden. Evidence-based guidelines for promoting cancer screening include the Guide to Community Preventive Services which recommends both client- and provider-oriented approaches to increase screening rates [6]. As in many areas of health care, however, there is a gap between evidence-based guidelines and actual practice [7]. In safety net health care systems, which include public hospitals, federally-funded community health centers and Federally Qualified Health Centers (FQHCs), local health department clinics, and free clinics that provide care for low-income, uninsured and vulnerable patients, this gap may be particularly wide. In community health centers, a centerpiece of the nation’s health care safety net, less than 35 % of patients aged 51 to 74 had been appropriately screened for colorectal cancer (CRC) in 2014; in contrast, 65.1 % of the general population in this age group was current on CRC screening in 2012 [4, 8].

Numerous theories and models have been proposed to inform and study the adoption and implementation of innovations, including evidence-based interventions such as recommendations to increase cancer screening [9–12]. The Consolidated Framework for Implementation Research (CFIR) attempts to advance our understanding of implementation across a range of settings and types of interventions by synthesizing and categorizing constructs across different theories and models [13]. The CFIR organizes 39 constructs and sub-constructs within five major domains: intervention characteristics, outer setting, inner setting, characteristics of the individuals involved, and the process of implementation. Much of the research using CFIR to date has been qualitative [14–21]. Some studies were intentionally designed to examine CFIR constructs [19] and others organized emerging themes using CFIR categories following data collection [22–24]. Damschroder and Lowery recently illustrated a methodology for using CFIR to qualitatively identify constructs that differentiate levels of implementation [14]. This or similar methodology has been used to examine implementation in several recent studies [14, 25, 26].

Relatively few studies have examined implementation of EBPs to increase cancer screening. A recent study used CFIR before actual implementation to inform the adaptation of an evidence-based program promoting CRC screening in a FQHC [7]. A second qualitative study of factors influencing cancer prevention in FQHCs did not use a conceptual model per se but identified competing priorities (e.g., medical home transformation), lack of reimbursement, and insufficient patient insurance as barriers to screening [27]. Federal reporting requirements were seen as a facilitator to improved cancer prevention and control. Neither of these studies identified factors that distinguished levels of implementation of EBPs for cancer screening.

The current study aims to fill such gaps by using CFIR to conduct a secondary analysis of data collected from an initiative to increase cancer screening in safety net settings. Understanding barriers and facilitators to cancer screening in settings that serve patients with significant health disparities is an important step in identifying strategies to decrease these disparities. Given persistent health disparities in cancer and the importance of accelerating adoption of EBPs to promote cancer screening, the American Cancer Society (ACS) developed the Community Health Initiatives: Community Health Advocates Implementing Nationwide Grants for Empowerment and Equity (CHANGE grants) program. This program funds primary care systems, with an emphasis on safety net providers, to implement EBPs to increase breast and/or colorectal cancer screening. Many of the grantees were FQHCs or FQHC look-alikes; the latter are certified as meeting federal health center requirements but are not part of the same funding stream. Through conducting a secondary analysis of data collected as part of an evaluation of the ACS’s Community Health Initiatives CHANGE Grants program, the current study aims to identify factors that distinguished implementation performance across safety net systems funded through this ACS initiative.

## Methods

### Primary evaluation

#### Program description

The ACS CHANGE Grants program promotes EBPs for cancer screening as recommended by the US Community Preventive Services Task Force [6] and the National Colorectal Cancer Roundtable [28]. Recommended client-oriented strategies include client reminders, small media, group education, one-on-one education, reduction of structural barriers, and reduction of out-of-pocket costs. Provider-oriented strategies include provider assessment and feedback, and provider reminder and recall systems. Grants in 2013 ranged from $40,000 to $80,000, with the majority funded at $50,000.

#### Study sample

Of the 68 grant recipients of the CHANGE program in 2013, nine health systems were selected for site visits. To maximize variation, systems were chosen based on level of implementation as indicated in interim progress reports, cancer site (breast vs. colorectal), priority population (e.g., race/ethnicity), geographic location, and corporate funders. Qualitative data were collected during site visits conducted 7–8 months into the 12-month grants. Semi-structured interviews were conducted with three to nine key informants per system and with ACS primary care managers. Leadership at each system identified all staff involved in project implementation and evaluators attempted to interview all identified staff. In some cases, interviews were conducted with two to three staff simultaneously, depending on staff availability, but the majority of interviews were conducted one-on-one. Key informants included executive directors, chief medical officers, information technology staff, quality improvement coordinators, medical assistants, nurses, community health workers, and patient navigators. A total of 52 interviews were conducted with 61 individuals for an average of six interviews per system.

#### Data collection instruments

##### Interview guides

Interview guides were developed by ACS evaluation staff, with tailoring for category of respondent (i.e., leadership, staff, and ACS primary care managers). The guides did not directly address CFIR constructs but focused on implementation with both general and specific questions surrounding implementation. Interview topics included intervention selection, start-up activities, implementation details (e.g., implementers, training, implementation processes), challenges and facilitators to implementation, policy and practice-level changes, staffing structure, partnerships, and sustainability. See Table 1 for a list of interview questions by topic. The interviews averaged 60 min in length and were audio recorded and transcribed verbatim.

**Table 1:** Key informant interview guide questions

Topic	Interview questions
Clinic characteristics	About how many patients does your clinic serve annually? About how many over the age of 50? Please describe services provided by the clinic. Is your clinic part of a larger health network?
ACS relationship	Describe your organization’s relationship with ACS prior to working with this project. What factors influenced your decision to work with ACS on this grant? How often did you meet/talk with ACS staff during the project period? What type of training or TA did ACS staff provide? What type of training or TA would be useful to assist you in implementing this type of intervention in the future?
Intervention selection	Were you involved in the decision-making process for choosing these interventions? Can you describe in detail what activities you are doing with the ACS project? How did you decide to implement these particular intervention strategies for this grant program? Who was involved in that decision? What factors influenced you to select these intervention strategies? If you had already been doing any of these interventions before the grant program, for how long? What factors influenced you to originally implement this intervention?
Intervention implementation	Please describe the steps you took to implement the intervention. What staff are involved in implementation? What are their titles and general responsibilities? What were their responsibilities related to the intervention? About what percentage of their time would you say is dedicated to the intervention? What training did staff receive prior to implementing the intervention? What type of ongoing training/meetings did you have during the project period to assess progress? Are community health workers involved in implementation? If this is a new intervention, how long did it take to fully implement the intervention? If this is an existing intervention, how long did it take to implement the project specific activities? Please describe your process for referring individuals for screening. What were some of the challenges in implementing the intervention? What steps did you take to overcome the challenges? What were some factors that helped you implement the intervention?
Program goals	Were you involved in developing the program goals and target numbers? What are your target number of screenings and individuals educated for the grant program? How did you determine the target numbers? Did you provide baseline screening referral data to ACS prior to this project? After starting this project? How did you determine your baseline? Were you able to reach your education goals? Screening goals? If not, what factors do you think prevented you from reaching your goals? If so, what factors helped you reach your goals? Do you know the targeted number of screenings and individuals to be educated for the project?
Data collection	How do you work with ACS to report data on education and screening numbers? How do you usually track these types of activities? What difficulties did you experience in providing the data to ACS? What would make it easier for you to report data to ACS?
Electronic health record utilization	Does your clinic use an electronic health record system? How do you use this EMR to obtain information on colorectal cancer screening? With regard to the project, to what extent did you utilize EMR? Were there any particular challenges utilizing EMR to support the intervention?
Sustainability	Will the partnership with ACS continue after the project ends? If so, in what ways? After the project is over, will the work implemented as a result of the project continue? If so, in what ways? Were any staff hired for the project? Will they remain on staff? With regard to the project, are there plans to advance the work implemented? If so, please explain.

##### Progress reports

Each system worked with local ACS support staff, to varying degrees, to develop annual goals and screening targets for their program. However, how such goals were developed varied. Some were based on baseline screening data; some had difficulty calculating their baseline screening rates and determined their goals based on professional judgment. Each system submitted quarterly reports to ACS through an online tracking tool as well as a final report. Grantees provided data to regional ACS primary care managers who then entered it into a centralized database. Data included screening information (screening targets, numbers screened) and intervention-specific data (number of contacts made though one-on-one education, outreach and client reminders).

### Secondary data analysis

#### Implementation level determination

We categorized the nine systems into high-, medium-, and low-performing systems based on data collected from the quarterly and final reports as well as the process and qualitative data collected from the site visits. Each system implemented a combination of patient- and provider-oriented strategies, with plans evolving as the grant year unfolded. To facilitate cross-system comparisons, we examined the extent to which client reminders were implemented. Client reminders was the only EBP implemented by all nine systems. We also examined the extent to which the systems attained their quantitative screening targets. Given the variable quality of reporting, we also included qualitative indicators of success in our analysis including potential sustainability of selected EBPs as described by the key informants at each site. Table 2 provides a brief description of each of the nine systems and summarizes how they performed on these dimensions.

**Table 2:** Description of systems and implementation outcomes by level of implementation

System and number of interviews	Cancer type and screening test	Description of system and patient volume (large, medium or small)^a^	Goal achievement for client reminders^b^	Goal achievement for screening test^b^	Level of implementation and likely sustainability
System A (*n* = 9)	CRC (FIT)	FQHC (medium)	Exceeded	Exceeded	High
			13,380 (161 % of goal)	FIT: 2130 (128 % of goal)	Sustainability likely due to workflow redesign and practice changes
				Colonoscopy: 170 (480 % of goal)	
System B (*n* = 8)	CRC (colonoscopy)	Community hospital and health system (large)	Exceeded	Exceeded	High
			3420 (140 % of goal)	3500 (143 % of goal)	Sustainability unclear—need funding for patient navigator
System C (*n* = 7)	CRC (colonoscopy)	University health system with FQHC partner	No goal	Exceeded	High
		(Large with small partner)	230	2100 (419 % of goal)	Sustainability unclear—dependent on volunteers and partnerships
System D (*n* = 6)	CRC (FIT)	FQHC (large)	Exceeded	Exceeded	Medium
			8680 (2480 % of goal)	260 (115 % of goal)	Sustainability unclear—vague discussion
System E (*n* = 6)	Breast (mammography)	FQHC (medium)	Exceeded	Did not meet goal	Medium
			1760 (352 % of goal)	970	Sustainability unclear—will need to scale back outreach
System F (*n* = 7)	Breast (mammography)	FQHC (small)	No target	Exceeded	Medium
			200	1180 (147 % of goal)	Sustainability mixed: EMR changes sustainable; patient navigator requires funding
System G (*n* = 5)	Breast (mammography)	Regional health system (large)	No target	Not met	Low
			350	250	Sustainability unlikely for screening—need funding & no concrete actions taken
System H (*n* = 3)	Breast and colorectal (mammography and colonoscopy)	FQHC (medium)	No report	No report	Low
					Sustainability mixed—EMR changes sustainable, outreach will be scaled back
System I (*n* = 6)	Breast (mammography)	FQHC look-alike (small)	No target	Not met	Low
			180	<5	Sustainability unclear-limited practice changes to sustain

^a^Large >40,000 patients; medium 15,000 to 40,000 patients, small <15,000 patients

^b^From final reports

#### Qualitative data analysis

Each safety net system is considered a case in our analysis. We employed a largely deductive approach using a codebook based on the CFIR constructs and definitions. All analysts (*N* = 6) participated in testing the codebook by coding two transcripts from one system and through multiple research team meetings to refine and reach consensus on code definitions and use. Analysts were instructed to adhere strictly to the definitions and apply codes without making inferences from the data. After the codebook was finalized, the qualitative data analysis was conducted in three phases.

The goal of the first phase was to organize the data with CFIR codes and build the foundation for case-based analysis [29]. A pair of analysts coded each transcript independently with CFIR codes and then met to resolve discrepancies. After consensus was reached, final codes were applied to the transcripts using NVivo 10. For each case, an analyst was designated as a case “expert” who coded all transcripts of that case and reviewed its project proposal and evaluation reports. All analysts kept brief memos for each transcript, which were compiled by the expert for each case to facilitate future analyses [30].

The goal of phase two was to distill the data into brief summaries for each CFIR construct and for each case. We generated a report via NVivo containing all relevant text units for each construct from all transcripts within each case. From this report, we created summaries and applied ratings for each construct first at the individual transcript level and then at the case level. We applied a rating system with two dimensions: magnitude and valence. “Valence” refers to the construct’s influence on implementation of EBPs—positive, negative, mixed, or largely descriptive with no valence (see Table 3). At the individual transcript level, valence was determined by respondents’ accounts related to the specific construct. At the case level, we also took into account whether or not the respondents in different transcripts agreed with each other in terms of the constructs’ influence on implementation. “Magnitude” refers to the extent to which the constructs were discussed. At the individual transcript level, “magnitude” was represented by the total number of mentions of a construct in a transcript and the proportion of text coded with that construct per transcript. At the case level, the “magnitude” was determined using two methods that complemented each other. It was first determined by multiplying the number of excerpts coded with a construct within a case by the proportion of transcripts that were coded with a construct per case. We also assessed “magnitude” based simply on the proportion of transcripts within a case that were coded with a construct (i.e., none = 0 %, few = 1 = 25 %, some = 26–50 %, many = 51 = 75 %, most = 76–99 %, all = 100 %).

**Table 3:** Construct rating criteria

Rating	Criteria
+	Facilitated the implementation of intervention
−	Hindered the implementation of intervention
+/−	Posed both positive and negative impact (mixed effects) on implementation
D	Purely descriptive, no impact upon implementation described

The case expert summarized the following information for each construct in a case-specific matrix with constructs as rows and transcripts as columns: (1) a brief summary of how this construct manifested in a particular transcript, (2) a magnitude and a valence rating of the construct’s influence on implementation of EBPs in a particular transcript. Once this step was completed for all transcripts within each case, the case expert reviewed the information across all the transcripts and aggregated them to the case level for each CFIR construct with a case-level summary and ratings. The case expert also composed a case narrative identifying the most salient constructs that affected implementation. Both the matrix and narrative for each case were reviewed by a secondary analyst. Discrepancies were noted by the second analyst and resolved through discussion.

The goal of phase three was to identify patterns of influence on implementation for each CFIR construct across all nine cases [29]. We sought to identify CFIR constructs that distinguished high-, medium-, and low-performing cases. To achieve this goal, the case summaries and ratings from phase two were imported into a case-ordered matrix in which cases were listed by level of implementation [31]. Two analysts independently reviewed the magnitude and the valence of all constructs across nine systems to identify distinguishing patterns across high, medium, and low-performing systems. Patterns were then confirmed by a third analyst. Table 4 presents results from the intervention characteristics domain as an example of our approach (see additional tables for the other domains).

**Table 4:** System-ordered matrix of magnitude and valence of intervention characteristics constructs by level of implementation

Construct	High level of implementation			Medium level of implementation			Low level of implementation			Distinguishing
Intervention characteristics	A	B	C	D	E	F	G	H	I	
Intervention source										
Magnitude	Some	Some	Few	None	Few	Some	None	None	Few	No
Valence	+/−	0	0	~	+	+	~	~	+	
Evidence strength and quality										
Magnitude	Few	Some	Some	None	None	Some	Some	None	Few	No
Valence	+	0	+	~	~	+	+	~	+	
Relative advantage										
Magnitude	Some	Some	Few	Some	Many	Some	Few	Some	None	No
Valence	+	+/−	+	+	+/−	+	+	+	~	
Adaptability										
Magnitude	Some	Many	Some	None	Few	All	Few	Some	Some	Yes
Valence	+	+	+	~	+	+	+	+	+	
Trialability										
Magnitude	Some	Some	Few	None	Some	Some	None	None	Few	Yes
Valence	+	+	0	~	+	+	~	~	0	
Complexity										
Magnitude	Some	Some	Few	Some	Few	Few	Few	None	None	No
Valence	+/−	−	−	−	−	0	−	~	~	
Design quality and packaging										
Magnitude	Some	Some	Many	None	Few	Some	Few	Many	Few	Borderline
Valence	+/−	+	+	~	+	+	−	+/−	+	
Cost										No
Magnitude	None	None	Many	Some	Few	Few	Few	Some	Few	
Valence	~	~	+	−	0	0	0	−	0	

Notes on magnitude: none = 0, few = 1 = 25 %, some = 26–50 %, many = 51 = 75 %, most = 76–99 %, all = 100 % of transcripts in each system that mentioned the construct. Notes on valence: ~ = not applicable, 0 = descriptive only, + = positive, − = negative, +/− = mixed

## Results

Table 2 briefly describes the nine systems and presents implementation outcomes by system. Six systems were FQHCs or FQHC look-alikes. The other three systems were large and complex health systems with varying primary care arrangements (e.g., FQHC partner, or affiliated primary care clinics). One of these was an urban community health system (system B), another was a large university-based health system (system C), and the third was a large regional health system (system G). Across the nine systems, four served over 40,000 patients (systems B, C, D, G), three served 15,000 to 40,000 patients (systems A, E, H), and two were relatively small with fewer than 15,000 patients (systems F and I). Four of the systems focused on EBPs for colorectal cancer screening (systems A, B, C, D), four on breast cancer screening (systems E, F, G, I), and one on both types of cancer (system H). The systems rated as having high implementation levels (systems A, B, C) completed more than 2000 screening tests in the funded grant year. Those rated as having medium implementation levels (systems D, E, F) reported more than 250 but fewer than 1500 screenings, combined with at least modest success in implementing client reminders. Systems with low levels of implementation (systems G, H, I) reported 250 screenings or fewer, and modest or few client reminders.

### Intervention characteristic domain

Among the eight constructs within this domain, we observed distinguishing patterns for two constructs: adaptability and trialability.

#### Adaptability

Adaptability, or the degree to which an intervention can be tailored to better fit the organizational context, was described as a positive influence in eight systems. The magnitude of influence, however, was stronger in the high-performing systems relative to the low-performing systems (see Table 4). In the three higher-performing systems, adaptations were made to the EBP delivery models after initial implementation based on lessons learned from early delivery challenges. For example, system A changed the implementer model (from receptionist to medical assistants) for carrying out patient reminders to increase efficiency and increase privacy for patients. System B changed the frequency and timing of the mailing and phone calls for conducting patient reminders to decrease the frequency of missed colonoscopy appointments, modified data-capturing tools, re-vamped how to deliver education on colonoscopy preparation, and eliminated extra steps for patients during the screening process:

*“… at one point a patient would go to tell them on the sixth floor that they had arrived, but then go down to the second floor to register at the cashier, and then go back to the sixth floor to say, okay, I checked in, now what? It was – so we try to cut all the extra steps and just make one smooth flow.”*

Two of the three medium-performing systems discussed adaptation. System E’s community health worker came up with creative ways to deliver patient education. In system F, comments about adaptation described how certain features of the intervention could be adapted, such as use of appropriate languages to educate patients and supplementing the current electronic medical record (EMR) system with other tracking tools. In contrast, there was less discussion of adaptation in the three low-performing systems. Respondents from systems G and I discussed patient education materials. A respondent from system H simply expressed appreciation for being able to adapt.

While adaptation manifested positively across systems, the main differences across systems lied in the magnitude to which adaptation was discussed and the types of adaptation being made. Detailed descriptions of adaptations were shared in high- and medium-performing systems and most of the adaptations were made to the delivery of the intervention. The lower-performing systems described fewer changes, and when they did, changes were primarily made to educational materials, thus suggesting that adaptability, or the actual adaptation of EBPs, influences implementation outcomes.

#### Trialability

Trialability, or the ability to pilot test before full-scale implementation, was discussed in six systems; three systems did not refer to this construct. Four out of six accounts of this construct were positive and two were descriptive (Table 4). The high-performing systems engaged in more discussion of trialability than medium- and low-performing systems, as reflected in the higher magnitude scores. All high-performing systems tried at least one pilot to figure out the best model for their system. For example, system A selected a clinic location that was viewed as ideal for piloting and determining the best approach to engage and remind patients about the fecal immunochemical test (FIT):

*So it’s kind of a fertile ground to play things out……we’re big enough, so just a great place to have pilots.*

Two of the medium-performing systems also used pilots. System E pilot tested the role of outreach workers and learned it was a struggle to schedule outreach events at churches and local pharmacies. System F tried different workflow procedures, such as communicating to providers via EMR pop-ups. Only one system from the lower-performing group (system I) mentioned trialability, and they described the entire CHANGE program as a pilot program with a lot of challenges, while also helping them identify areas for improvement. Because the higher-performing systems described trialability in significant detail and it was not discussed or only mentioned in general terms in the lower-performing systems, trialability appears to have an important influence on implementation. This construct was closely associated with adaptability, given that adaptations were often made after trying a particular approach to implementation.

### Outer setting domain

A large majority of respondents across all nine cases discussed patient needs and resources and cosmopolitanism as important factors influencing the implementation of EBPs (see Additional file 1: Table S1). However, discussion of these and other outer setting constructs did not vary noticeably by level of implementation.

### Inner setting domain

Three inner setting constructs clearly distinguished systems by implementation outcomes (Additional file 1: Table S2): leadership engagement, tension for change, and access to information and knowledge.

#### Leadership engagement

Leadership engagement, a sub-construct under readiness for implementation, was referenced in all systems. The accounts of this construct described how leaders were involved in the implementation of the EBPs, and included establishing program goals and individual roles, providing ongoing support and guidance, providing direct services for patients, and monitoring progress with feedback to staff. Eight out of nine systems described this factor as exerting a positive impact. However, the magnitude of influence appeared higher among the high-performing systems. In the high-performing systems, all respondents shared positive views. For example, in system A, both the front-line navigators and staff leaders thought that the program director played a key role in pulling things together to make the CHANGE program happen:

*“So she (program director) was the main driver of the project, and …kind of showed us what the – how we would benefit from this, how important it is.”*

System B described how executive-level leaders were at the table asking questions and ensuring accountability through reporting of outcomes. Leadership engagement was mentioned less frequently across the medium- and low-performing systems, with fewer concrete examples of how leaders were actively engaged.

These patterns were repeated in the initial phases of the implementation process. In all high-performing systems, both the leadership and key implementers were able to present the CHANGE program or the EBPs in a way that was relevant and useful, which helped them earn buy-in from providers and staff for actual implementation. Such communication typically occurred during staff or leadership meetings, in which project leaders presented the benefits and rationale for the program with supporting data and evidence. In contrast, two of the lower-performing systems expressed uncertainty about the project at the onset rather than enthusiasm. Because the discussions of leadership engagement varied both in terms of magnitude and depth across performance groups, combined with varying approaches to “selling” the program, leadership engagement appears to have an important influence and distinguish implementation outcomes.

#### Tension for change

Tension for change, or the degree to which stakeholders perceive the current situation as intolerable or needing change, was discussed in eight out of nine systems. The account of this construct was often described in the context of identifying gaps in quality measures (e.g., low cancer screening rates), deficiencies in reaching patients (e.g., existing outreach only reached a limited number of patients), and problems with completing cancer screenings (e.g., high no-show rates for colonoscopies or low return rate of FIT kits). Such gaps and problems were often identified through quality improvement efforts and were frequently reported as the rationale for participating in the CHANGE program. The construct was most commonly discussed (with higher magnitude) in the high-performing systems with detailed and specific descriptions of why the program was needed. For example, a respondent from system B shared how the program addressed a specific need identified by providers:

*“The oncologist and GI specialists approached us, this is a need that …. We’re having a lot of no-shows for colonoscopies. Can you guys help us? Usually when a specialist approaches and tells you, it’s real.”*

Respondents from the medium-performing systems also discussed tensions for change. System D respondents discussed challenges associated with getting accurate data on screening rates given the need to get reports back from specialists and enter them into EMR in a way that allows for report generation. System E discussed their low screening rates relative to neighboring FQHCs. In contrast, lower-performing systems either did not discuss any tension for change or made general observations about how the program provided an opportunity to address cancer. This pattern suggests that identifying specific deficiencies in measures and practice might have created more tension for change, leading to increased momentum for implementing the EBPs.

#### Access to information and knowledge

Access to information and knowledge refers to the ease of access to information about the intervention and how to incorporate it into organizational processes. Descriptions centered on training and educational resources available for the appointed implementers of the CHANGE program. Most accounts were positive among the high-performing systems, with adequate trainings for staff often cited as key to successful implementation. Training was not discussed as frequently in medium- and lower-performing systems and tended to be descriptive. Negative perspectives referred to a lack of consensus on training needs and the absence of written plans or protocols to guide implementation. For example, a respondent from system H described:

“*So there was no set process. There wasn’t a written down set process, which was – it made it more difficult……when you’re on your own, it can be kind of overwhelming sometimes to make sure that you hit everything that you need to do.*”

Given the notable differences in access to information and availability of training in the higher-performing systems relative to the lower-performing systems, it appears that this construct facilitated successful implementation. This construct also appeared to be highly related to leadership engagement given the knowledge and information for implementation was most often initiated and provided by organizational leaders.

### Individual characteristics domain

Constructs from the individual characteristics domain were not often discussed across the nine systems (Additional file 1: Table S3). Knowledge and beliefs about the intervention and personal attributes were discussed most commonly, but none of the constructs from this domain differentiated systems by levels of performance.

### Implementation process domain

Respondents discussed the process of implementing the EBPs in depth, with substantive discussions focused on planning, executing, and reflecting and evaluating. Despite the salience of key constructs within this domain, only formally appointed internal implementation leaders varied by level of implementation (Additional file 1: Table S4).

#### Engaging formally appointed internal implementation leaders

Formally appointed internal implementation leaders, a sub-construct under engaging, was discussed by all nine systems. Implementation leaders were typically patient navigators, health educators, nurses, or medical assistants who implemented the major tasks of the CHANGE program in each system. High- and medium-performing systems tended to discuss the importance of these roles more than lower-performing systems. The magnitude of influence of this construct was higher among high- and medium-performing systems. For example in system A, the medical assistants were appointed as primary implementers of the CHANGE program to increase CRC screening, which improved workflow and required less direct involvement of the providers:

“*We basically taught the MAs how to do the bulk of the work and then when they got to either a question or at that point at all, once – if they had a FIT test and it was obvious it was done in the last year, then the MA knew not to order it. But if there was no FIT test, the MA ordered it.”*

Systems B and C also described the roles of implementation leaders in depth, with respondents from both systems describing the specific roles and responsibilities of patient navigators in EBP implementation. In contrast, respondents from system D did not discuss a single point person and system E shared that no new staff were hired, but clinical leadership were very engaged in the entire intervention planning and implementation process. Lower-performing systems tended to describe implementation leaders less often and in neutral terms, or in the case of system G, acknowledging the challenges associated with not having a dedicated point person:

*“But we knew going in from the beginning that it was something that was going to be a challenge because we didn’t have a dedicated staff member. We couldn’t take somebody out of clinical time to send them out to the community.”*

These differences in the presence of a formally appointed implementation leader across performance levels, combined with the more frequent and detailed discussions in higher-performing systems, suggest that this construct differentiates levels of implementation.

## Discussion

This study examined factors influencing implementation of EBPs to promote breast and colorectal cancer screening in safety net systems using CFIR. One of our main findings was that leadership engagement clearly distinguished high-, medium-, and low-performing systems. Our findings are consistent with a number of studies that have identified leadership engagement as key to successful implementation [32–34]. Using similar methods in a study focused on implementation of a weight loss program into five VA systems, Damschroder and Lowery found that leadership engagement was strongly associated with successful implementation; leaders allocated staff time, solved problems, obtained needed resources, and kept the program visible [14]. In contrast, a recent study on implementation of internet-based patient-provider communication by Varsi and colleagues used similar methods and did not find leadership engagement to be consistently related to implementation [25]. Such variance in the influence of leadership across different study settings suggests that while leadership engagement is generally considered key to success, how to maximize leaderships’ positive influence in implementation might depend on the nature of the setting and EBP.

In addition to leadership engagement, formally appointed implementation leaders strongly distinguished level of implementation in our study. Within the VA study, most of the systems had a strong coordinator, and thus, formal implementation leaders did not distinguish between systems due to lack of variability [14]. In the Varsi et al. study, formally appointed implementation leaders weakly distinguished level of implementation [25]. Our findings are consistent with a qualitative study by Martinez-Gutierrez et al., which found designation of non-physician staff to do specific prevention activities had a major impact on cancer screening rates in a single FQHC system [35]. Taken together, our findings on leadership engagement and formally appointed implementation leaders suggest that successful implementation of EBPs requires leaders at multiple levels to function with high capacity. Leaders at the executive level should be highly engaged in goal setting, establishing roles, getting buy-in from providers, providing training, and monitoring progress, while front-line implementation leaders are crucial in day-to-day operations and pilot testing, as well as identifying inefficiencies in current processes and suggesting changes for program delivery [36].

We identified four additional constructs that distinguished levels of implementation across systems; two of these constructs were from the intervention characteristics domain: adaptability and trialability. Higher-performing systems in our study gave detailed accounts of how they adapted or tailored the intervention to fit their setting; systems that struggled with implementation tended to describe adaptations to educational materials rather than clinic flow or processes. Higher-performing systems in our study also conducted pilot studies of the EBPs to inform broader implementation, often using a quality improvement process. Interestingly, neither of these constructs were distinguishing factors in the Damschroder and Lowery study due to lack of variation across systems for adaptability, and because none of the systems conducted a trial of the intervention. Trialability was a distinguishing factor in the Varsi et al. study [25]. Overall, our findings on the importance of various aspects of the intervention itself are consistent with the large body of research on diffusion of innovations and related studies that have examined how intervention characteristics influence implementation [11, 34, 37, 38].

Two additional inner setting constructs distinguished levels of implementation in our study, including tension for change and access to information and knowledge. Similar to prior studies, systems with higher levels of implementation tended to describe a concrete need for the program [25]. In our study, needs tended to be identified through quality improvement efforts showing room for improvement in screening rates or challenges with existing screening approaches. Access to information and knowledge usually related to training opportunities provided by leadership in our study, with high-performing systems describing trainings more often and in greater detail. Training was not available as part of the VA implementation project, and training was viewed as adequate in all of the systems in the Varsi et al. study [14, 25]. Other studies have similarly identified training of key staff as important for implementation [16, 33]. With respect to the other inner setting constructs, Sohng et al. found that communication and resource availability were positively associated with the likelihood of EBP implementation for cancer screening [39]. We did not find these constructs to distinguish levels of implementation, in large part because network and communication appeared to have similar influence (in terms of magnitude and valence) across the nine systems.

Another notable finding from our study is that, despite variability among the nine safety net systems in terms of size, population served, and organizational infrastructure, none of these factors differentiated implementation levels. In a systematic review that included cancer screening efforts in both FQHCs and other health organizations, Anhang Price et al. identified organizational factors and processes that helped to increase cancer screening rates at each step in the cancer screening process [36]. Interestingly, similar to our findings, organizational structures, such as size and type, were not associated with screening rates. Rather, improvements in screening rates were largely driven by organizational strategies to (1) limit the number of interfaces across organizational boundaries; (2) recruit patients, promote referrals, and facilitate appointment scheduling; and (3) promote continuous patient care. These organizational processes were similar to those described in our study and correspond to the roles of leadership and formally appointed implementation leaders, which were both distinguishing factors for implementation success.

Our study employed a slightly different rating approach than the rating system proposed by Damschroder and Lowery and the CFIR guide [14]. The “valence” dimension in both approaches was essentially the same and characterizes the influence of CFIR constructs as positive, negative, mixed, and neutral/descriptive. The “strength” dimension in Damschroder’s and Lowery’s rating system is generally comparable to our “magnitude” dimension. However, their “strength” rating system incorporated several distinct factors, including the level of agreement among participants, strength of language, and use of concrete examples. We used level of agreement among participants to inform our “valence” dimension (e.g., did participants agree on whether the construct had a positive or negative influence), and we found it challenging to assess strength of language in a systematic manner with multiple respondents within each case and multiple mentions of constructs within each transcript. Thus, we chose to assess magnitude as described above, which factored in the extent to which a particular construct was discussed across respondents from each site.

### Limitations

Several limitations should be considered when assessing the performance of each system and our related findings. The first limitation is related to the accuracy of the quantitative measures of completed screenings, which might in turn impact the accuracy of implementation outcomes. The participating systems used a variety of methods to specify their screening targets, and to some extent these were arbitrarily determined. Targets were not set using a standardized formula, and grantees were not provided specific criteria for targets. Some systems set goals using baseline data from previous years but baseline data did not always reflect the system’s actual performance due to reporting and EMR system errors. All these limitations, however, reflect the reality of implementation challenges in resource-constrained safety net health care settings. Nevertheless, it does have implications for the validity of our implementation outcome categories, despite our efforts to triangulate across measures. Second, the systems that performed better in terms of implementation outcomes tended to be addressing CRC rather than breast cancer. This may reflect that systems ready to take on CRC were with higher capacity given that addressing CRC within FQHCs is relatively new compared to efforts to increase breast cancer screening. Third, the interview guide was developed to answer ACS’s evaluation questions, and was not informed by CFIR. As a result, some of the constructs that did not emerge as salient may have been salient if asked about explicitly. On the other hand, the interview guides were designed to identify barriers and facilitators to implementation with mostly general questions not specifically addressing CFIR constructs. Therefore, the themes that emerged from the responses to these general questions were in essence perceived by the participants to be the most salient factors. Though the study was not originally designed to test CFIR, the fact that so many of these emergent factors identified in this post hoc, deductive fashion were consistent with CFIR domains and constructs could serve to validate the utility of CFIR [34]. As a meta-theoretical framework, CFIR was developed to serve as a comprehensive typology of factors influencing implementation, to compare results across contexts and to identify new theoretical developments. A recent systematic review of the use of CFIR found that the majority of published studies were using CFIR to guide data analysis alone [40]. Our study will add to this repository of studies that could facilitate cross-context comparisons. Future research could compare findings between studies using CFIR to guide data analysis alone and those using CFIR throughout the data collection and analysis process.

## Conclusions

Overall, our study suggests that despite the resource-constrained environment, with moderate support, such as the grants and technical assistance from ACS, safety net health systems can successfully implement EBPs to increase cancer screening. While size, patient population, and organization structure did not distinguish implementation success, the key factors to successful implementation may include strong leadership and front-line implementation leaders, adequate training and information, and organizational processes to identify deficiencies, pilot test new ideas, and make adaptations accordingly. Future interventions aiming to increase cancer screening or implement other EBPs in similar safety net settings could consider intervening on these factors while designing their efforts. Practitioners implementing EBPs could consider focusing their resources on developing implementation strategies that target these factors, such as training designated implementation leaders and setting up organizational processes to provide information and ongoing feedback to those involved in day-to-day implementation. Our study also contributes to the growing body of research on CFIR. As described by Damschroder and Lowery [14], contributing to a repository of findings will enable the field to begin to understand which constructs have predictive ability, which can be manipulated for better implementation outcomes, and the situations in which specific constructs are salient. Our study also provides suggestions for exploring relationships among CFIR constructs which will aid in future studies of construct validity.

## Additional file

Additional file 1.
**Table S1.** Site-ordered matrix of magnitude and valence of outer setting constructs by level of implementation. **Table S2**. Site-ordered matrix of magnitude and valence of inner setting constructs by level of implementation. **Table S3**. Site-ordered matrix of magnitude and valence of individual characteristics constructs by level of implementation. Table S4. Site-ordered matrix of magnitude and valence of implementation process constructs by level of implementation. (DOCX 21 kb)

## Abbreviations

ACS: American Cancer Society
CFIR: Consolidated Framework for Implementation Research
CHANGE: Community Health Advocates Implementing Nationwide Grants for Empowerment and Equity
CRC: Colorectal Cancer
EBP: Evidence-based Practice
EMR: Electronic Medical Record
FIT: Fecal Immunochemical Test
FQHC: Federally Qualified Health Center
MA: Medical Assistant
