# Erratum to: ‘Integrating evidence-based practices for increasing cancer screenings in safety net health systems: a multiple case study using the Consolidated Framework for Implementation Research’

**DOI:** 10.1186/s13012-016-0494-3

**Published:** 2016-09-23

**Authors:** Shuting Liang, Michelle C. Kegler, Megan Cotter, Emily Phillips, Derrick Beasley, April Hermstad, Rentonia Morton, Jeremy Martinez, Kara Riehman

**Affiliations:** 1 Emory Prevention Research Center, Department of Behavioral Sciences and Health Education, Rollins School of Public Health, Emory University, Atlanta, USA; 2 Statistics and Evaluation Center, Department of Intramural Research, American Cancer Society, Inc., Atlanta, USA

## Erratum

Unfortunately, the original version of this article [1] contained an error. The author Emily Phillip’s names were interchanged. They have been corrected in this erratum and there will also be an update to amend this error. The author’s given name is Emily and their family name is Phillips.

## References

[1] Liang S, Kegler M, Cotter M, Phillips E, Beasley D, Hermstad A, Morton R, Martinez J, Riehman K (2016). Integrating evidence-based practices for increasing cancer screenings in safety net health systems: a multiple case study using the Consolidated Framework for Implementation Research. Implement Sci..

