# Supplementary material for: Integrating evidence-based practices for increasing cancer screenings in safety net health systems: a multiple case study using the Consolidated Framework for Implementation Research
Source: Implement Sci. 2016 Aug 2;11:109. doi: 10.1186/s13012-016-0477-4 (PMC4970264; doi:10.1186/s13012-016-0477-4)
Supplement: Additional file 1. — Table S1. Site-ordered matrix of magnitude and valence of outer setting constructs by level of implementation. Table S2. Site-ordered matrix of magnitude and valence of inner setting constructs by level of implementation. Table S3. Site-ordered matrix of magnitude and valence of individual characteristics constructs by level of implementation. Table S4. Site-ordered matrix of magnitude and valence of implementation process constructs by level of implementation. (DOCX 21 kb) [file 13012_2016_477_MOESM1_ESM.docx]

**Table S1. Site-Ordered Matrix of Magnitude and Valence of Outer Setting Constructs by Level of Implementation**

| **Construct** | **High Level of Implementation** | | | **Medium Level of Implementation** | | | **Low Level of Implementation** | | | **Distinguishing** |
| --- | --- | --- | --- | --- | --- | --- | --- | --- | --- | --- |
| **Outer Setting** |  |  |  |  |  |  |  |  |  |  |
| Patient Needs & Resources  Magnitude  Valence | Most  +/- | Many  +/- | All  + | All  +/- | Most  +/- | All  0 | All  + | Many  0 | Most  +/- | No |
| Cosmopolitanism  Magnitude  Valence | Some  + | Many  + | Most  +/- | All  +/- | All  + | Most  + | All  + | Many  + | Some  + | No |
| External Policies & Incentives  Magnitude  Valence | Few  - | Few  0 | Some  + | Many  - | Few  0 | Many  0 | Some  + | Some  + | Some  0 | No |
| Other Characteristics  Magnitude  Valence | Few  0 | None  ~ | Some  +/- | Some  - | Most  +/- | None  ~ | Some  + | Some  0 | Few  + | No |

Note: Peer pressure was not discussed in the interviews and therefore not included in this table.

**Table S2.** **Site-Ordered Matrix of Magnitude and Valence of Intervention Inner Setting Constructs by Level of Implementation**

| **Construct** | **High Level of Implementation** | | | **Medium Level of Implementation** | | | **Low Level of Implementation** | | | **Distinguishing** |
| --- | --- | --- | --- | --- | --- | --- | --- | --- | --- | --- |
|  | **A** | **B** | **C** | **D** | **E** | **F** | **G** | **H** | **I** |  |
| Structural Characteristics  Magnitude  Valence | Many  +/- | Most  +/- | Many  - | All  +/- | Many  +/- | Most  0 | Most  + | All  0 | Some  + | No |
| Networks & Collaborations  Magnitude  Valence | Most  + | Most  +/- | Many  +/- | All  + | Most  + | Most  + | All  + | Many  + | Most  + | No |
| Communication  Magnitude  Valence | Many  +/- | Some  +/- | Some  + | Some  +/- | Many  + | Some  + | Many  + | Many  + | Few  + | No |
| Culture  Magnitude  Valence | Few  +/- | None  ~ | Few  + | Few  + | Many  + | Some  + | Few  + | Many  + | Few  + | No |
| Tension for Change  Magnitude  Valence | Few  + | Many  + | Some  + | Few  0 | Few  0 | Some  + | Few  + | None  ~ | Few  + | Yes |
| Compatibility  Magnitude  Valence | Most  + | Many  + | Many  + | Some  +/- | Most  + | Most  + | Most  + | Many  + | Some  + | No |
| Relative Priority  Magnitude  Valence | Many  +/- | Some  + | None  ~ | Some  + | Few  + | Some  0 | Some  + | None  ~ | Many  + | No |
| Organizational Incentives & Rewards  Magnitude  Valence | Some  + | None  ~ | None  ~ | None  ~ | Few  + | Few  + | None  ~ | None  ~ | None  ~ | No |
| Goals and Feedback  Magnitude  Valence | Few  + | Some  + | Some  0 | None  ~ | Few  + | Many  + | Few  0 | Some  0 | None  ~ | No |
| Learning Climate  Magnitude  Valence | Few  + | Some  + | None  ~ | None  ~ | Some  + | Some  + | Some  + | None  ~ | None  ~ | No |
| Leadership Engagement  Magnitude  Valence | Most  + | Many  + | Many  + | Some  + | Many  + | Some  +/- | Some  0 | Many  + | Some  + | Yes |
| Available Resources  Magnitude  Valence | Most  + | Some  + | Many  +/- | Many  +/- | Most  + | All  +/- | Some  + | All  +/- | Many  + | No |
| Access to Knowledge and Information  Magnitude  Valence | Some  +/- | Many  + | Many  + | Few  +/- | Some  + | Few  0 | Few  0 | None  ~ | Few  - | Yes |

**Table S3. Site-Ordered Matrix of Magnitude and Valence of Individual Characteristics Constructs by Level of Implementation**

| **Construct** | **High Level of Implementation** | | | **Medium Level of Implementation** | | | **Low Level of Implementation** | | | **Distinguishing** |
| --- | --- | --- | --- | --- | --- | --- | --- | --- | --- | --- |
|  | **A** | **B** | **C** | **D** | **E** | **F** | **G** | **H** | **I** |  |
| Knowledge & Beliefs  Magnitude  Valence | Some  0 | Few  0 | All  0 | Some  0 | Most  + | Some  + | Many  0 | Many  0 | Some  + | No |
| Self-Efficacy  Magnitude  Valence | None  ~ | Some  + | Some  + | None  ~ | Few  + | None  ~ | None  ~ | Some  + | Some  + | No |
| Stage of Change  Magnitude  Valence | Few  0 | None  ~ | None  ~ | None  ~ | Few  + | None  ~ | None  ~ | Some  0 | None  ~ | No |
| Identification with Organization  Magnitude  Valence | Few  0 | None  ~ | None  ~ | None  ~ | None  ~ | None  ~ | None  ~ | None  ~ | None  ~ | No |
| Personal Attributes  Magnitude  Valence | Some  + | Many  + | All  + | Many  + | All  + | Some  0 | Most  + | Some  + | Many  +/- | No |

**Table S4.** **Site-Ordered Matrix of Magnitude and Valence of Intervention Process Constructs by Level of Implementation**

| **Construct** | **High Level of Implementation** | | | **Medium Level of Implementation** | | | **Low Level of Implementation** | | | **Distinguishing** |
| --- | --- | --- | --- | --- | --- | --- | --- | --- | --- | --- |
|  | **A** | **B** | **C** | **D** | **E** | **F** | **G** | **H** | **I** |  |
| Planning  Magnitude  Valence | Many  + | Many  + | Many  0 | Most  +/- | Most  + | Many  + | Some  + | Many  + | Some  + | No |
| Engaging  Magnitude  Valence | Few  + | Some  + | None  ~ | None  ~ | None  ~ | None  ~ | Few  0 | None  ~ | None  ~ | No |
| Formally appointed internal implementation leaders  Magnitude  Valence | Many  0 | Many  + | Many  +/- | Few  + | Many  + | Most  + | Some  0 | Some  + | Few  0 | Yes |
| Champion/  Opinion Leaders  Magnitude  Valence | Few  + | Few  + | Few  + | None  ~ | None  ~ | Some  + | Few  + | None  ~ | Few  + | No |
| External Change Agents-ACS  Magnitude  Valence | Few  +/- | Some  + | Most  + | Some  +/- | All  + | Some  0 | Most  + | Many  + | Many  + | No |
| External Change Agents-non-ACS  Magnitude  Valence | None  ~ | None  ~ | Some  +/- | None  ~ | Few  +/- | Some  + | Few  + | None  ~ | Some  + | No |
| Executing  Magnitude  Valence | Most  0 | Many  +/- | Many  +/- | Most  +/- | Most  +/- | Most  + | Most  +/- | All  0 | Many  - | No |
| Reflecting & Evaluating  Magnitude  Valence | Many  +/- | Many  + | All  +/- | All  +/- | Many  +/- | Most  +/- | Most  + | All  0 | Many  0 | No |
